# Multifunctional Gold Nano-Cytosensor With Quick Capture, Electrochemical Detection, and Non-Invasive Release of Circulating Tumor Cells for Early Cancer Treatment

**DOI:** 10.3389/fbioe.2021.783661

**Published:** 2021-11-11

**Authors:** Rui Zhang, Qiannan You, Mingming Cheng, Mingfeng Ge, Qian Mei, Li Yang, Wen-Fei Dong, Zhimin Chang

**Affiliations:** ^1^ School of Biomedical Engineering (Suzhou), Division of Life Sciences and Medicine, University of Science and Technology of China, Hefei, China; ^2^ CAS Key Laboratory of Biomedical Diagnostics, Suzhou Institute of Biomedical Engineering and Technology, Chinese Academy of Science (CAS), Suzhou, China; ^3^ College of Life Science and Biotechinology, Mianyang Teachers’ College, Mianyang, China; ^4^ Chongqing Institute of Green and Intelligent Technology, Chinese Academy of Sciences, Chongqing, China; ^5^ Jinan Guokeyigong Science and Technology Development Co., Ltd, Jinan, China

**Keywords:** CTCs, early diagnosis and treatment, electrochemical cytosensor, non-invasive release, multifunctional Au nanoparticles

## Abstract

Circulating tumor cells (CTCs) are metastatic tumor cells that shed into the blood from solid primary tumors, and their existence significantly increases the risk of metastasis and recurrence. The timely discovery and detection of CTCs are of considerable importance for the early diagnosis and treatment of metastasis. However, the low number of CTCs hinders their detection. In the present study, an ultrasensitive electrochemical cytosensor for specific capture, quantitative detection, and noninvasive release of EpCAM-positive tumor cells was developed. The biosensor was manufactured using gold nanoparticles (AuNPs) to modify the electrode. Three types of AuNPs with controllable sizes and conjugated with a targeting molecule of monoclonal anti-EpCAM antibody were used in this study. Electrochemical impedance spectroscopy (EIS) and differential pulse voltammetry (DPV) of the cytosensors were performed to evaluate the cell capture efficiency and performance. The captured 4T1 cells by the AuNPs hindered electron transport efficiency, resulting in increased EIS responses. The cell capture response recorded using EIS or DPV indicated that the optimal AuNPs size should be 17 nm. The cell capture response changed linearly with the concentration range from 8.0 × 10 to 1 × 10^7^ cells/mL, and the limit of detection was 50 cells/mL. After these measurements, glycine-HCl (Gly-HCl) was used as an antibody eluent to destroy the binding between antigen and antibody to release the captured tumor cells without compromising their viability for further clinical research. This protocol realizes rapid detection of CTCs with good stability, acceptable assay precision, significant fabrication reproducibility with a relative standard deviation of 2.09%, and good recovery of cells. Our results indicate that the proposed biosensor is promising for the early monitoring of CTCs and may help customize personalized treatment options.

## Introduction

Because malignant tumor cells are derived from solid tumors and are present in peripheral blood, circulating tumor cells (CTCs) are critical to cancer metastasis (Mostert et al., 2009; Schindlbeck et al., 2009; Gradilone et al., 2011; Hristozova et al., 2011; Xie et al., 2015). The rapid and sensitive detection of CTCs in the peripheral blood is an effective and appropriate approach for the diagnosis of cancer patients (Paterlini-Brechot and Benali, 2007; Mostert et al., 2009; Mostert et al., 2012; Alix-Panabières and Pantel, 2013; Shahneh, 2013). Moreover, the isolation of CTCs from the peripheral blood has significant clinical implications because it can provide smart and personalized treatment (Hong, 2013; Souza e Silva et al., 2014; J Alvarez-Cubero et al., 2016; Miyamoto et al., 2016; Tan et al., 2017; Liu et al., 2018). Therefore, the development of a dynamic technique for the sensitive detection and noninvasive release of pathogenic cells is necessary.

Recently, the development of sensitive and quantitative detection of CTCs has attracted extensive research attention (Denis et al., 1997; Nakanishi et al., 2000; Wen et al., 2014; Shinden et al., 2015). Traditional cancer detection methods primarily include flow cytometry (Hu et al., 2010), reverse-transcriptase polymerase chain reaction (Zhao et al., 2011), and immunohistochemistry (Gogoi et al., 2016). Although the above methods performed well in terms of sensitivity and precision, they are expensive and time consuming in addition to requiring complex instruments, hampering their application in biological analysis and clinical diagnosis. Accordingly, the development of a low-cost, sensitive, and simple method for the detection and efficient release of captured CTCs is necessary and has considerable significance in the early diagnosis and monitoring of the relevant cancer processes.

Presently, academic attention has shifted toward electrochemical biosensors that utilize noninvasive and highly sensitive nanomaterials (Thévenot et al., 2001; Wang, 2005 a; b; 2006; Peng et al., 2020). For example, by combining aptamer and gold-magnetic core-shell nanoparticles, Khoshfetrat fabricated an aptamer-based electrochemical biosensor with a nitrogen-doped graphene-modified electrode. This aptasensor exhibited a linear response over a wide dynamic range of leukemia cancer cells, ranging from 10 to 1 × 10^6^ cells/mL (Khoshfetrat and Mehrgardi, 2017). Pang synthesized a cytosensing device based on ZnO nanodisks@g-C3N4 quantum dots for the detection of CCRF-CEM cells ranging from 20 to 2 × 10^4^ cells/mL (Pang et al., 2018). These studies demonstrate the advantages of using metal nanoparticles as a design strategy for electrochemical biosensors. Appropriate nanomaterials must be selected for the manufacture a new generation of electrochemical biosensors. Compared with other nanoparticles, gold nanoparticles (AuNPs) provide an ideal binding site for biomolecules, which is beneficial for electron transfer to the electrode. Such characteristics have been widely used to fabricate electrochemical biosensors with improved performance (Zhong et al., 2013; Gikunoo et al., 2014; Qian et al., 2019). However, few studies have investigated the effect of the size of the AuNPs on enhancing the detection performance because the size of AuNPs may affect the analysis results. Cancer cells overexpress specific proteins, which are often considered to be specific markers in CTC analytical technology for different types of cancer cells (Moskaluk et al., 2002). For example, the epithelial cell adhesion molecule (EpCAM), which is overexpressed in most epithelial tumor cells, is often identified as a specific biomarker for epithelial tumor cells (Moskaluk et al., 2002).

**SCHEME 1:**
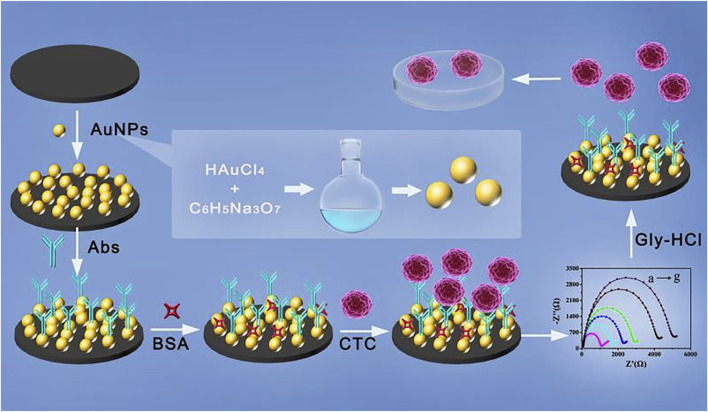
Stepwise procedure of the electrochemical cytosensor for cell capture, detection, and release.

In the present study, cytosensors based on AuNPs of different sizes were used for the electrochemical detection of CTCs. The AuNPs attached on the surface of the glassy carbon electrode (GCE) act as a good electron conductor that facilitates electrochemical cytosensing. Subsequently, the EpCAM antibody (anti-EpCAM) was modified on the surface of AuNPs to enhance the specificity of EpCAM-positive tumor cells. The prepared anti-EpCAM/AuNPs electrode was specific for tumor cell anchoring. The adhesion of tumor cells on the surface of the electrode could alter the electron transfer resistance, thereby allowing the formation of an impedance-based cytosensor. We investigated the effect of the size of the AuNPs on the detection performance of the fabricated cytosensors. Electrochemical impedance spectroscopy (EIS) and differential pulse voltammetry (DPV) were used to assess the capability of the fabricated cytosensors with different particle sizes (17, 30, and 50 nm). For the cytosensor fabricated with 17 nm AuNPs, the widest range of 4T1 cell detection (8.0 × 10 to 1 × 10^7^ cells/mL) was obtained with a lower LOD of 50 cells/mL, which was considerably lower than that of the other two fabricated cytosensors. As a control, HeLa and J774A.1 cells with low EpCAM expression were also applied under the same experimental conditions. The glycine hydrochloride (Gly-HCl) buffer introduced into the cytosensor system acted as an eluent that could break the combination within the antibody–antigen to release the trapped cells (Scheme 1). In this study, the developed cytosensor had high specificity for EpCAM-positive CTCs, good stability, biocompatibility, significant fabrication reproducibility, and noninvasive release. Thus, the developed cytosensor has promising application prospects in the early clinical diagnosis and treatment of cancer.

## Methods

### Synthesis of AuNPs

Gold seeds were prepared using a standard method. Briefly, 1 ml of HAuCl_4_ (25 mM) was mixed with sodium citrate (150 ml, 2.2 mM) and heated for 1 h. Then, the obtained solution was used as the seed solution. After synthesis, the solution was cooled to 90°C, HAuCl_4_ solution (1 ml, 25 mM) was added, and the reaction was completed after 30 min. By repeating these steps, a series of AuNPs with gradually increasing particle sizes was obtained (Bastús et al., 2011). The nanoparticles were labeled as G0, G1, and G2. Finally, the AuNPs were characterized using UV-visible absorption spectrum (UV), TEM, and XPS.

### Assembly Process of the Electrochemical Cytosensor

The assembly process of the AuNP-based electrochemical cytosensor is shown in Scheme 1. First, the GCE (3 mm) was polished with 0.05 μm alumina slurries and washed alternately with ethanol and water until a shiny mirror surface was obtained. Afterward, the mercapto functional group was modified on the electrode by cyclic voltammetry (CV). The potential range of CV detection was set from 0.0 to 1.4 V with a scan rate of 50 mV/s, and several circles were scanned in a 1 mM aqueous solution of mercaptoethylamine until the peak current value was stable (Moskaluk et al., 2002). After the reaction, the surface-modified electrodes were washed with PBS buffer (pH 7.4). Subsequently, the electrodes were immersed in the AuNPs solution (1 ml) overnight, and the AuNPs were tightly fixed on the surface of the GCE via gold and sulfhydryl bonds. Then the electrodes were rinsed with PBS buffer (pH 7.4). Subsequently, Nafion (5 μL, 0.05% in alcohol) was dropped onto the GCE surface to prevent leakage of the AuNPs. After 2–3 washings with PBS, the AuNP/GCE electrode was fabricated. AuNPs with different particle sizes were modified on the electrode surface using the same method. For immune modification, the AuNPs/GCE electrode was immersed in anti-EpCAM solution (60 μM) and then incubated in a refrigerator at 4°C for 4 h. Then, after washing with PBS, the electrode was immersed in BSA (1%, 50 μL) for 1 h to block nonspecific binding sites. Finally, the cytosensors for immunological recognition of EpCAM-positive CTCs were obtained.

### Cell Culture

4T1 cells, HeLa cells, and J774A.1 cells were cultured in a cell incubator at 37°C with 5% CO_2_. 4T1 cells were cultured in RPMI-1640 medium with 10% FBS and 1% penicillin–streptomycin. After incubation, the 4T1 cells were digested with 0.25% trypsin solution. The cell sediments were redispersed in PBS (pH 7.4), and the number of cells was determined using a hemocytometer counter.

### Cell Detection

For cell detection, the as prepared cytosensor was immersed into the cell suspension and incubated in a cell incubator for 2 h. After cell adsorption, the electrode was washed with PBS to remove unadsorbed cells. Finally, the prepared immunoelectrode acted as the working electrode, whereas Ag/AgCl and Pt wire electrodes were used as the reference and auxiliary electrodes, respectively. The electrode was inserted into a 0.1 M KCl, 5 mM K_4_ [Fe(CN)_6_], and 5 mM K_3_ [Fe(CN)_6_] solution. The impedance and current responses were recorded using EIS and DPV, respectively. The frequency range of the EIS detection was 0.1–10^5^ Hz. The range for DPV detection was from 0 to 0.6 V, with a 50 mV pulse amplitude. The potential range of CV detections was set from –0.6 to 0.6 V with a scan rate of 50 mV/s. Meanwhile, a 10 × 10 mm indium tin oxide (ITO) substrate after mercaptosilanization pretreatment was prepared as previously described process for the modified GCE electrode and cell detection. The captured cells were analyzed by fluorescence microscopy after DAPI staining.

### Cell Release

For cell release, the electrode with cell immobilization was immersed in Gly-HCl solution (0.1 M) for 30 s, and then several drops of 0.4 M NaOH solution were added to the Gly-HCl solution. The released cells were washed with PBS, centrifuged, and collected and cultured for further clinical analysis.

### Cell Viability Analysis

Cell viability was determined using Hoechst/PI staining assay. Briefly, Hoechst/PI solution containing 10 μg/ml Hoechst and 10 μg/ml PI in PBS was added to the cells and incubated for 5 min. The cells were washed and observed using a fluorescence microscope. The released cells were then incubated and observed under a microscope. The AuNPs were tested using a WST-1 assay to verify cytotoxicity. The working concentration of AuNPs was adjusted from 5.0 to 150.0 μM, and 4T1 cells were plated in 96-well plates and incubated for 24 h at 37°C. To evaluate cell viability, a plate reader was used to measure absorbance at 450 nm.

### Blood Samples

Blood samples were obtained from the healthy mice. The animal experimental protocols were approved by the Ethics Committee for the Use of Experimental Animals of Chinese Academy of Science. To verify the performance of the cytosensors in the simulated samples with leukocyte, the healthy blood sample was centrifugated at 1,500 rpm for 5 min and the cells deposit were then resuspended in 1 ml of red blood cell lysis buffer incubated for 2 min, and the precipitate was washed with PBS and disperse with 1 ml of PBS. Subsequently, the as prepared leukocyte samples mixed with 4T1 cells at concentrations of 1 ×10^2^, 1 ×10^3^, 1 ×10^4^, and 1 ×10^5^ cells/mL to prepare the simulated samples with leukocyte.

## Results and Discussion

### Characteristics of the AuNPs/GCE Electrode

**FIGURE 1:**
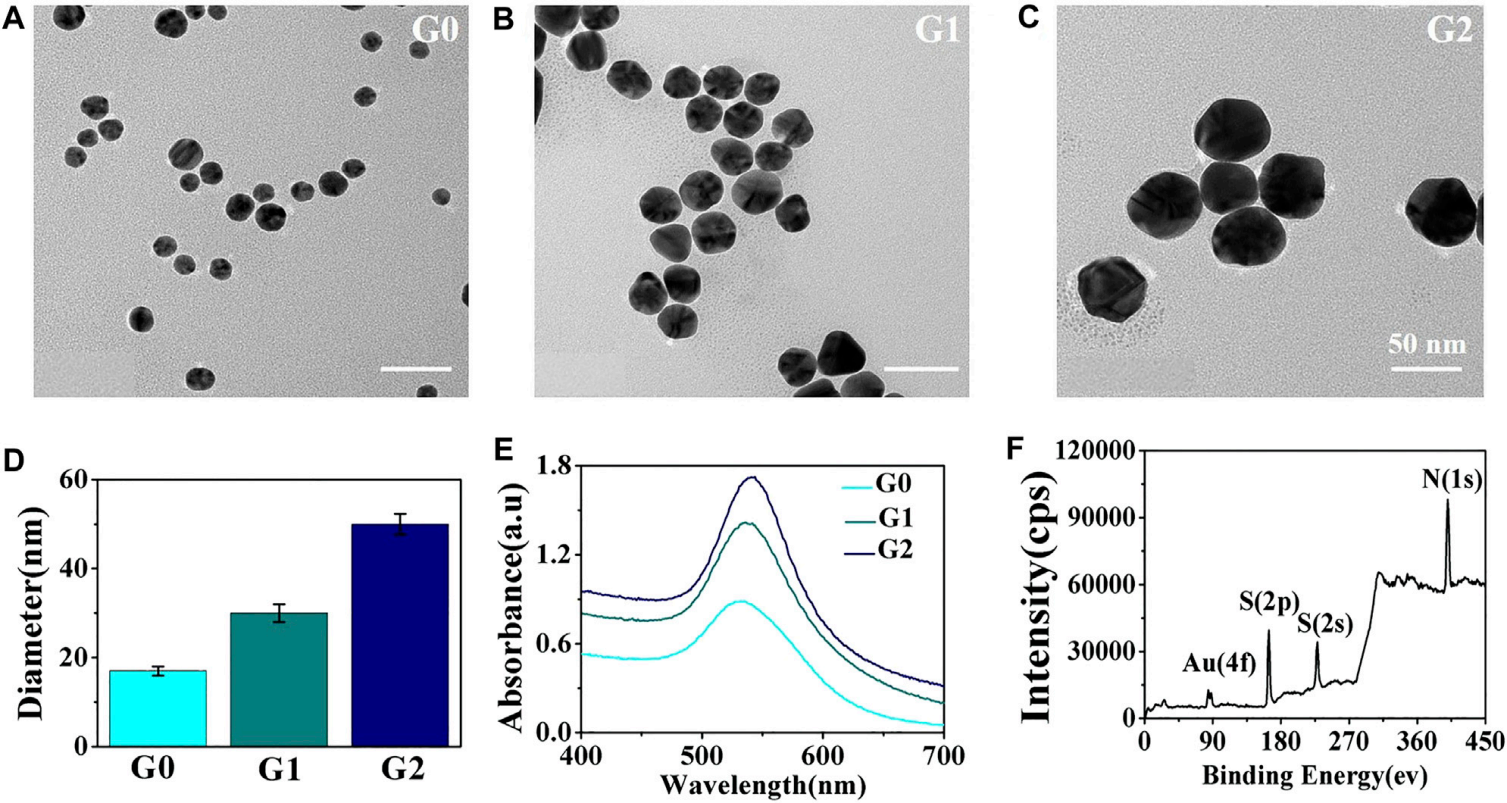
TEM images of the prepared **(A)** G0, **(B)** G1, and **(C)** G2. **(D)** Histogram of the prepared AuNPs sizes. **(E)** UV−vis spectra of the prepared AuNPs. **(F)** XPS characterization of the AuNPs/GCE electrode.

The morphology of the AuNPs was characterized. The TEM images in Figure 1 indicate that the synthesized AuNPs had a spherical shape with good monodispersity and uniform morphology. The average sizes of the AuNPs were 17 nm (G0), 30 nm (G1), and 50 nm (G2) (Figure 1D). Figure 1E shows the UV-visible absorption spectrum of the prepared AuNP water solution with an absorption peak at ∼535 nm. As shown in Figure 1F, the XPS results of the AuNP/GCE electrode revealed that the AuNPs were assembled onto the GCE electrode surface with Au-S bonds to form a self-assembled AuNP/GCE electrode.

**FIGURE 2:**
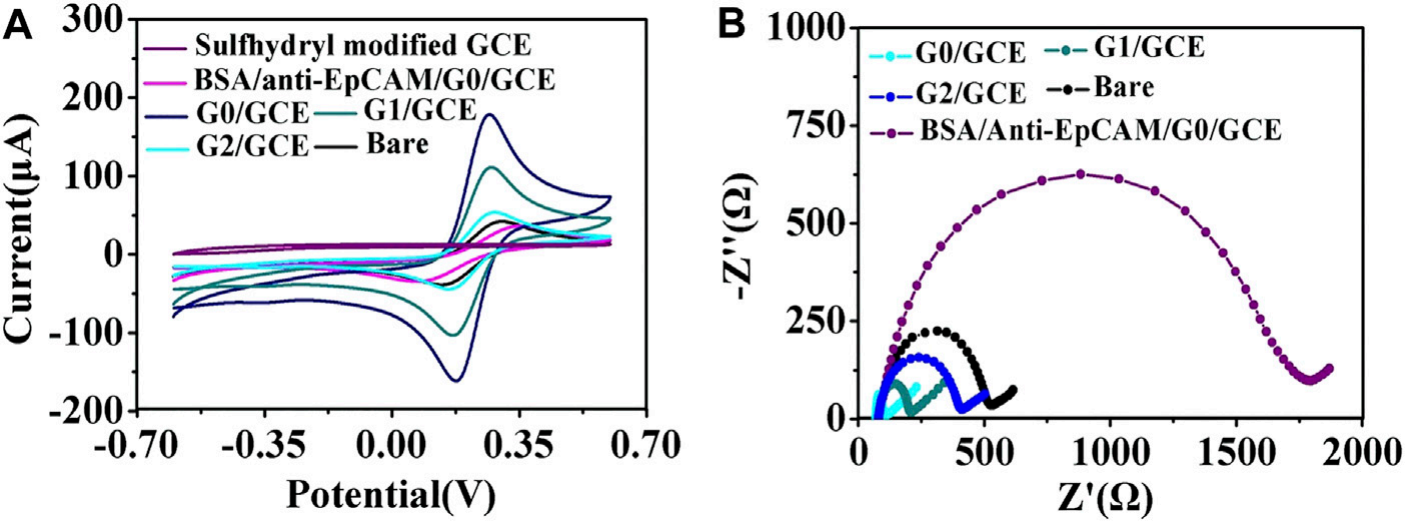
**(A)** CV curves of the AuNPs/GCE fabricated using AuNPs of different sizes. **(B)** EIS of the GCE modified by AuNPs with different sizes: (G0) 17 nm, (G1) 30 nm, and (G2) 50 nm.

For the AuNP/GCE electrodes fabricated using AuNPs of different sizes, CV and EIS were applied to monitor the electrode fabrication steps. The CV results are shown in Figure 2A. There was an evident increase in the oxidation peaks of the GCE electrodes modified by the AuNPs in comparison with the unmodified GCE electrode. In addition, as the size of the AuNPs increased, the well-defined oxidation current peaks of the [Fe(CN)_6_]^4−/3−^ redox couple gradually decreased, indicating that the 17 nm AuNPs could provide the highest improvement in charge transfer. Therefore, 17 nm AuNPs were used under the following optimal conditions for the fabrication of the cytosensor. It is well known that the impedance spectrum consists of a semicircular portion (the high-frequency process related to electron-transfer-limited, the value recorded as R_ct_) and a linear portion (the low-frequency process related to diffusion-limited). As demonstrated in Figure 2B and Supplementary Table S1, the R_ct_ of the AuNPs/GCE electrode was smaller than that of the GCE electrode (R_ct_ = 456.7 Ω), revealing a lower electron-transfer resistance and a faster charge exchange of [Fe(CN)_6_]^4-/3-^ on the AuNPs/GCE electrode in comparison to the bare GCE electrode. The R_ct_ values of the AuNPs/GCE electrodes fabricated by different AuNPs were 25.8 Ω (G0), 122.4 Ω (G1), and 319.9 Ω (G3) This shows that the impedance of the constructed electrode gradually increased as the particle size increased. After anti-EpCAM was covalently linked onto the surface of G0/GCE, the R_ct_ value and current changed significantly, which could be attributed to the weakly conductive properties of the anti-EpCAM molecule.

**FIGURE 3:**
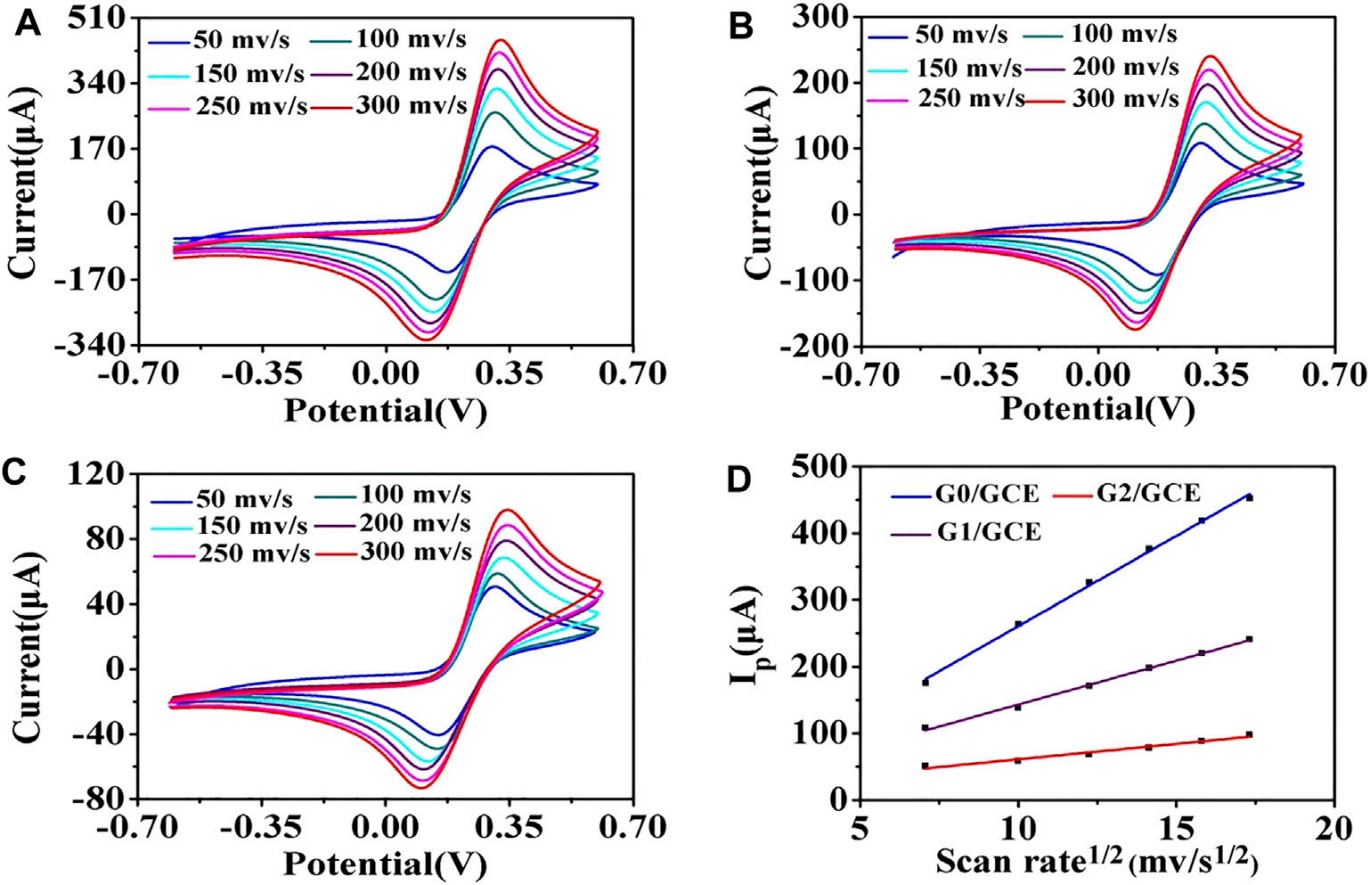
CV of the AuNP/GCE electrodes at different scan rates: 50, 100, 150, 200, 250, and 300 mV/s. **(A)** G0/GCE, **(B)** G1/GCE, **(C)** G2/GCE, **(D)** Randles–Sevcik plots of G0/GCE, G1/GCE, and G2/GCE electrodes.

Additionally, the morphologies of the AuNP/GCE slides fabricated with different sizes of AuNPs were characterized using SEM. As seen in Supplementary Figure S1, as the size decreased, the density of particles on the surface of the GCE increased, which may result in a higher efficiency of electron transfer on the electrode surface for AuNPs with good conductivity. The AuNP/GCE electrodes were tested by CV at different scan rates. As demonstrated in Figure 3D, the anodic peak current value increased significantly as the AuNP size decreased, indicating that G0/GCE had the largest electron transfer efficiency on the electrode surface, which is consistent with the above conjecture.

### Fabrication of Electrochemical Cell Sensor

**FIGURE 4:**
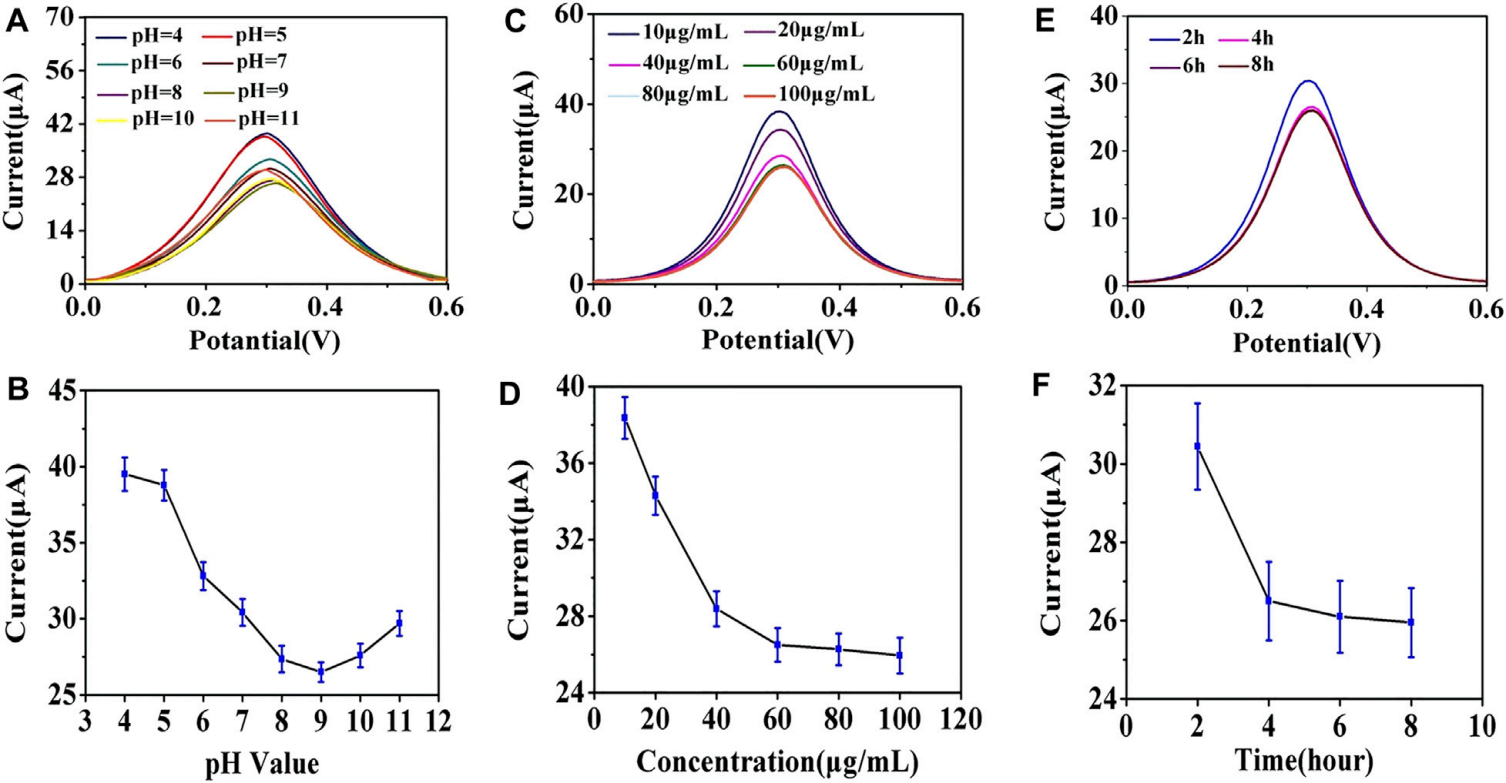
**(A)** Effect of anti-EpCAM-modified pH value on DPV response. **(B)** line graph of the effect of the pH value on the peak current value of DPV. **(C)** effect of anti-EpCAM concentration on DPV response. **(D)** line graph of the effect of anti-EpCAM concentration on the peak current value of DPV. **(E)** effect of anti-EpCAM binding time on the DPV response. **(F)** line graph of the effect of anti-EpCAM binding time on the peak current value of DPV.

To achieve optimal detection performance, several parameters in the electrochemical immunoassay were explored, including the optimal pH value of antibody modification, concentrations, and incubation time of anti-EpCAM conjunction. The G0/GCE electrode was used in subsequent procedures. The pH value of the working solution has a significant influence on the binding strength between antibodies and AuNPs, and it was adjusted using K_2_CO_3_ or HCl in this study. As shown in Figures 4A,B, when the pH was adjusted from 4.0 to 9.0, the corresponding DPV signal decreased rapidly, and when the pH value exceeded 9.0, the DPV signal increased with the value. This could be attributed to the fact that the strongest covalent binding strength of the antibodies we used and AuNPs occurred in an alkaline environment (pH = 9.0) (Stonehuerner et al., 1992; Sardar et al., 2009), and the optimal pH value was determined to be 9.0.

The concentration of anti-EpCAM was a critical factor in the construction strategy because it directly affected the binding efficiency of the cells. As shown in Figures 4C,D, the DPV signals decreased as the anti-EpCAM concentration increased, and a minimum CV response was obtained at the concentration of 60 μg/ml, which revealed the saturation modification of anti-EpCAM. The 60 μg/ml of anti-EpCAM is recommended for further research.

Incubation times of the antibodies and AuNPs were also explored. Figures 4E,F illustrated the influence of the DPV signal with different incubation time. The current response saturated at 4 h, and the optimal incubation time was 4 h.

### Electrochemical Characterization of Immuno-Cytosensor

Once the AuNP/GCE electrode surface antibody modification with the optimal experimental parameters was performed, the electrode was washed with PBS and blocked the nonspecific binding sites by BSA solution (1%, 50 μL) for 1 h. In the aforementioned process, the EIS method was utilized to monitor the different modification steps of the electrode for immediate and sensitive response to a change in the electrode surface (Quan et al., 2001). Supplementary Figure S2 and Supplementary Table S1 show the detailed changes in R_ct_ values using [Fe(CN)_6_]^4−/3−^ as the redox probe with anti-EpCAM and BSA modification. The results revealed that the R_ct_ value of the cell sensor was larger than that of the GCE electrode (R_ct_ = 456.7 Ω), indicating the weak conductive properties of the antibody. The cytosensors fabricated by the AuNPs with different sizes and modified by antibody were successfully assembled, and R_ct_ was 1,605.7 Ω (G0), 1,216.1 Ω (G1) and 940.4 Ω (G2).

### Detection Performance of the Electrochemical Cytosensor

**FIGURE 5:**
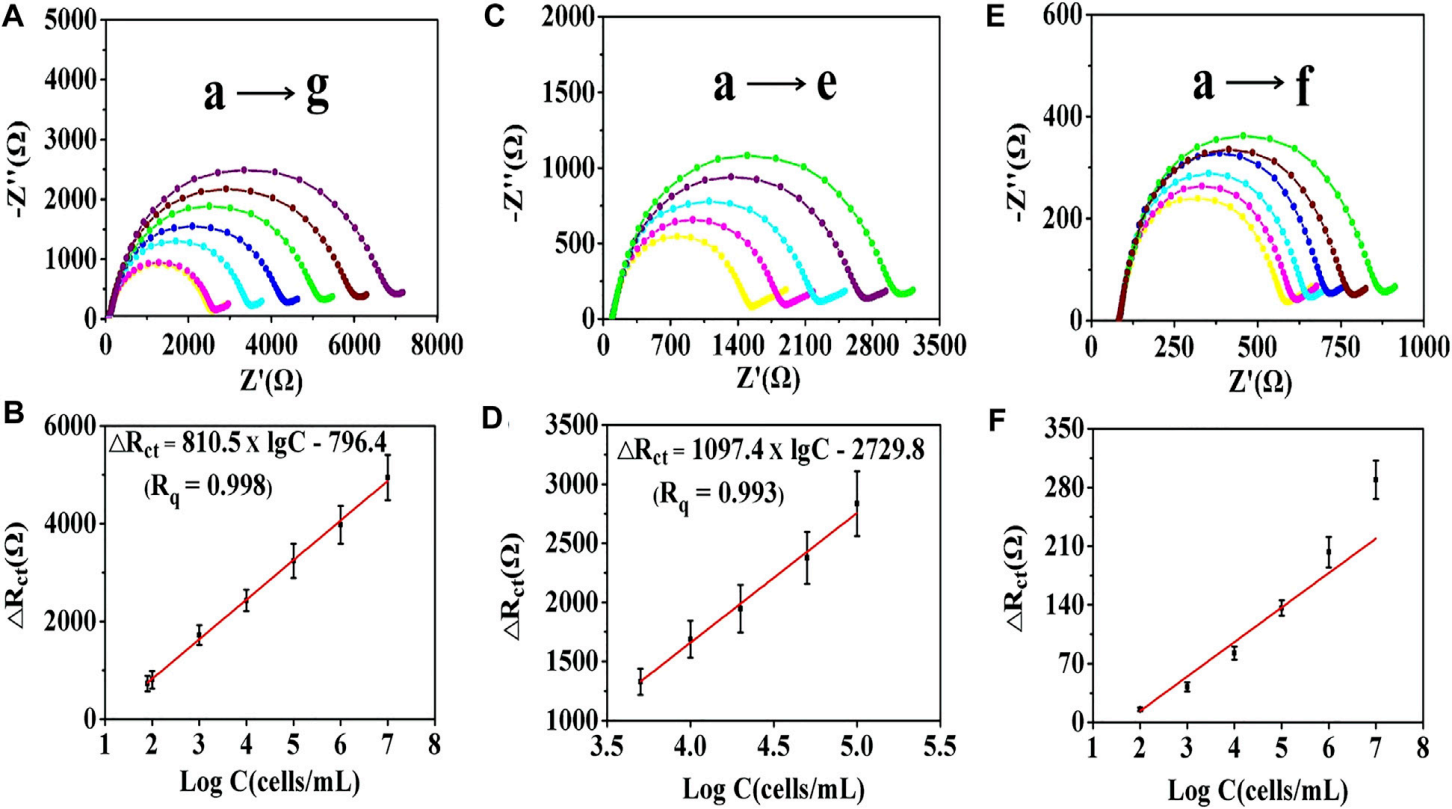
EIS curves of the cell sensors after capturing different concentrations of 4T1 cells. **(A)** EIS curves of the G0 cytosensor. **(B)** linear relationship between the impedance value of the G0 cytosensor and the logarithm of cells concentration: (a) 8.0 × 10, (b) 1.0 × 10^2^, (c) 1.0 × 10^3^, (d) 1.0 × 10^4^, (e) 1.0 × 10^5^, (f) 1.0 × 10^6^, and (g) 1.0 × 10^7^ cells/mL. **(C)** EIS curves of the G0/GCE electrode. **(D)** linear relationship between the impedance value of the G0/GCE electrode and the logarithm of cells concentration: (a) 5.0 × 10^3^, (b) 1.0 × 10^4^, (c) 2.0 × 10^4^, (d) 5.0 × 10^4^, and (e) 1.0 × 10^5^ cells/mL. **(E)** EIS curves of the GCE electrode and **(F)** linear relationship between the impedance value of the GCE electrode and the logarithm of cells concentration: (a) 1.0 × 10^2^, (b) 1.0 × 10^3^, (c) 1.0 × 10^4^, (d) 1.0 × 10^5^, (e) 1.0 × 10^6^, and (f) 1.0 × 10^7^ cells/mL. The error bars represent the standard deviation of three independent measurements.

In the present experiment, the cytosensor was incubated with a 4T1 cells solution for 2 h at 37°C (Supplementary Figure S3). 4T1 cells ranging from 10 to 10^7^ cells/mL were monitored for 2 h to estimate the ability of the electrochemical cytosensor. After washing three times with PBS, the EIS and DPV methods were used for the quantitative determination of tumor cells. For the EIS method, as shown in Figure 5 and Supplementary Figure S4, the R_ct_ values increased with the cell concentration owing to the trapped cells hindering the electron transfer. For the G0 AuNP cytosensor, when the cell concentration ranged from 8.0 × 10 to 10^7^ cells/mL, the equation ΔR_ct_ (Ω) = 810.5 × lgC -796.4 (*R* ^2^ = 0.998) was applied to record the relationship between 4T1 cell concentration (C) and impedance change value (ΔR_ct_). As an LOD, 55 cells/mL (S/N = 3) of 4T1 cells could be detected by EIS. The detection equations of the G1 and the G2 AuNPs cytosensors were ΔR_ct_ (Ω) = 598.1 × lgC - 754.5 (2.0 × 10^2^–10^6^ cells/mL, LOD = 112 cells/mL, *R* ^2^ = 0.997) and ΔR_ct_ (Ω) = 316.4 × lgC - 517.1 (5.0 × 10^2^–5.0 × 10^5^ cells/mL, LOD = 500 cells/mL, *R* ^2^ = 0.995) respectively (Supplementary Figures S4A–D). This revealed that the G0 AuNP cytosensor was optimal for the quantitative detection of tumor cells. For the G0 AuNP cytosensor, when the DPV method was adopted, as shown in Supplementary Figures S4E,F, the current response (I) decreased linearly with increasing logarithm values of the target cell concentrations within the range of 8.0 × 10 to 10^7^ cells/mL. The equation was I = −2.3 × lgC +22.1 (*R* ^2^ = 0.996), and the LOD was 50 cells/mL (S/N = 3). Compared with other methods that were used for quantitative analysis of CTCs, the aforementioned assay revealed significant detection sensitivity and lower LOD (Supplementary Table S2) (Zhang et al., 2010; Zhu et al., 2012; Cao et al., 2017; Bolat et al., 2021).

G0/GCE and bare GCE electrodes were employed to investigate their self-sensing properties. As shown in Figure 5C, for the G0/GCE electrode, the Nyquist diagrams changed proportionally to the logarithm of cells concentration with the range from 5.0 × 10^3^ to 1.0 × 10^5^ cells/mL. The linear relationship can be depicted as ΔR_ct_ (Ω) = 1097.4 × lgC - 2729.8 (*R* ^2^ = 0.993), and the LOD was 5,000 cells/mL (Figure 5D). For the bare GCE electrode, there was a minor response between the ΔR_ct_ value and the logarithm of the cell concentration (Figures 5E,F). The obtained analytical performance of the G0/GCE sensor could give the credit to the good conductivity of the AuNPs (Supplementary Figure S5). After covalent coupling of the anti-EpCAM, EpCAM-positive tumor cells could be specifically captured on the surface of the electrode. Compared with the bare AuNPs/GCE cytosensor, the immunocytosensor has excellent EIS detection capability, which is largely due to the highly specific antigen–antibody immunoreaction.

**FIGURE 6:**
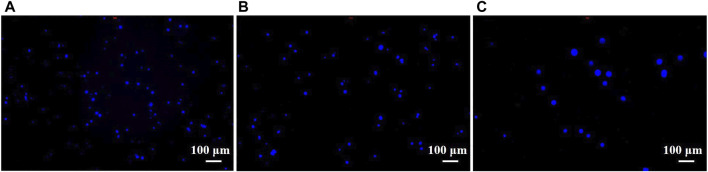
Fluorescence images of 4T1 cells captured by cell sensors. 4T1 cells captured by **(A)** G0, **(B)** G1, and **(C)** G2 cytosensors.

To analyze the number of captured tumor cells, a 10 × 10 mm ITO substrate after mercaptosilanization pretreatment was prepared by the same procedure and then incubated with 4T1 cells (1 × 10^5^ cells/mL) in PBS for 2 h. After the cleaning step with PBS, the captured cells were stained with DAPI for 20 min and detected using a fluorescence microscope. As shown in Figure 6, the number of cells captured by the constructed cytosensor decreased with the increase in the particle size of the AuNPs. This could be attributed to the fact that with the decrease in the particle size of the AuNPs, more AuNPs with larger surface area were modified onto the surface of GCEs, resulting in better capture performance of the sensor.

### Specificity and Reproducibility of the Electrochemical Cytosensor

**FIGURE 7:**
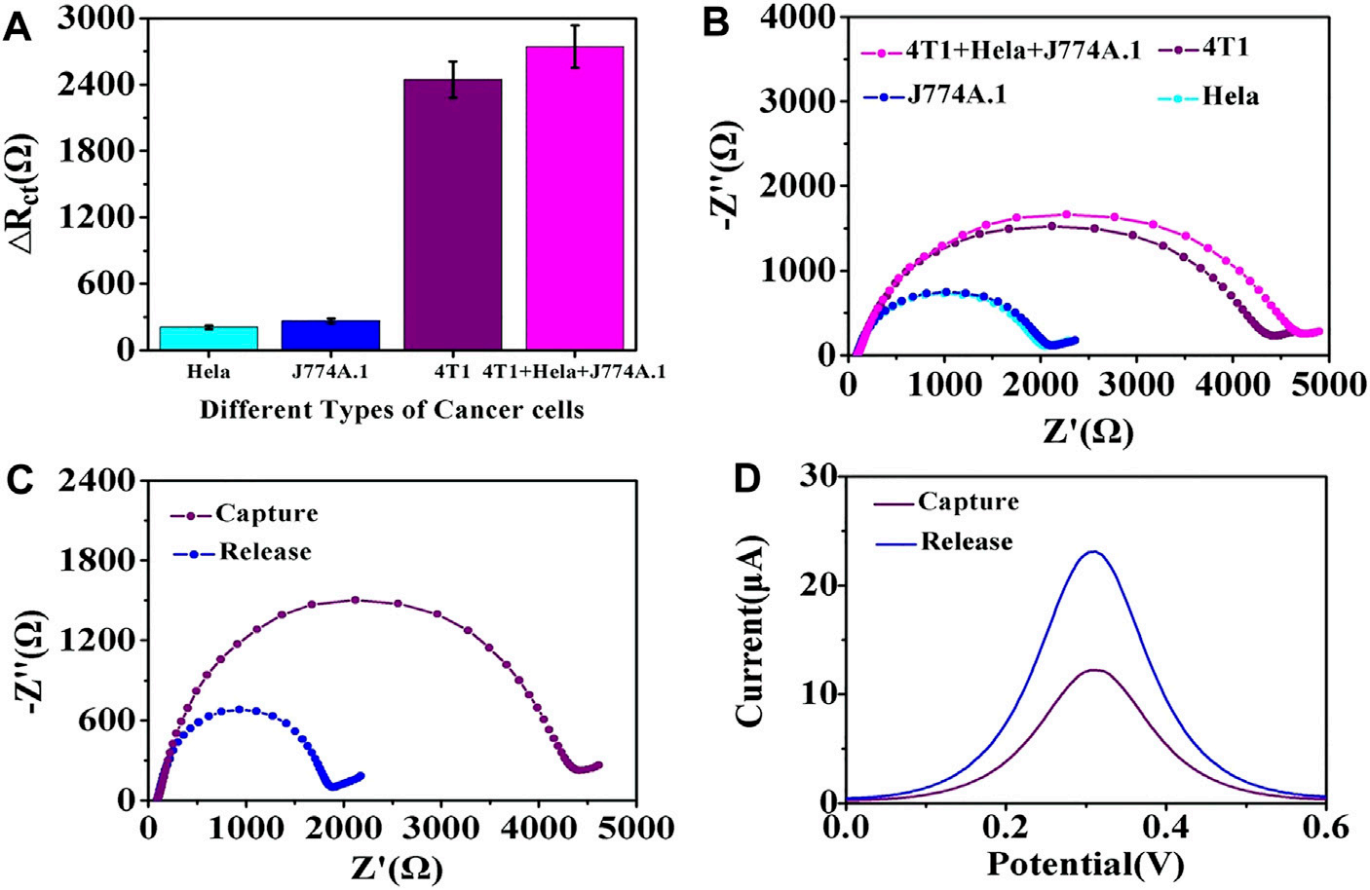
**(A)** Specificity of the proposed cytosensing strategy, with the ΔR_ct_ value of the G0 cytosensor after incubation with HeLa, J774A.1, or 4T1 cells measured in a solution containing [Fe(CN)_6_]^3−/4−^ (5 mM, 1:1) and 0.1 M KCl. The error bars represent the standard deviation of five replicate determinations. **(B)** typical EIS responses after capturing different types of cancer cells. **(C)** comparison of the EIS results and **(D)** comparison of the DPV results of the cytosensor after the capture of cells and after the release of cells.

To assess the specificity of the immunecytosensor, two EpCAM-negative cell lines, HeLa and J774A.1, were selected as controls. The cell lines (1.0 × 10^4^ cells/mL) were co-incubated with the fabricated cytosensor for 2 h and investigated via EIS. Figures 7A,B show that only EpCAM-positive 4T1 cells showed a noticeable R_ct_ change, whereas the EpCAM-negative cells only showed a slight increase in the R_ct_ value. Furthermore, the R_ct_ value of the mixture sample (1.0 × 10^4^ cells/mL of 4T1 cells, HeLa cells, and J774A.1 cells) was slightly higher than that of 4T1 cells, which could be due to the nonspecific adsorption of EpCAM-negative cells, revealing that the construction strategy results in significant selectivity for EpCAM-positive 4T1 cells.

The electrode reproducibility in the aforementioned method was determined using five freshly fabricated electrodes to detect the target cells five times at the same concentrations (10^4^ and 10^5^ cells/mL), with relative standard deviations of 2.09 and 3.15% (Supplementary Table S3), respectively. These data indicate the excellent reproducibility of this method.

Stability was also investigated as a vital property of the fabricated cytosensor. When the G0 cytosensor was stored at 4°C for 7 and 15 days, 96.5 and 92.1% of the initial current responses for 10^5^ cells/mL 4T1 cells were retained, respectively. These data suggest that this method exhibited good stability.

### Cell Release and Cell Viability Analysis

The release of captured target cells will facilitate subsequent biological analyses. After the Gly-HCl eluent destroyed the binding between the antigen and antibody, the captured 4T1 cells were released into the solution from the surface of the cytosensor. As shown in Figure 7C, the impedance of the electrode decreased after treatment with the Gly-HCl eluent and the release of captured cells. Similarly, the DPV results (Figure 7D) also reveal that the current signal of the electrode significantly increased after the release procedure. These results were due to the release of nonconductive cell entities from the electrodes and elimination of the electronic transmission screen, which led to the recovery of electrochemical signals.

**FIGURE 8:**
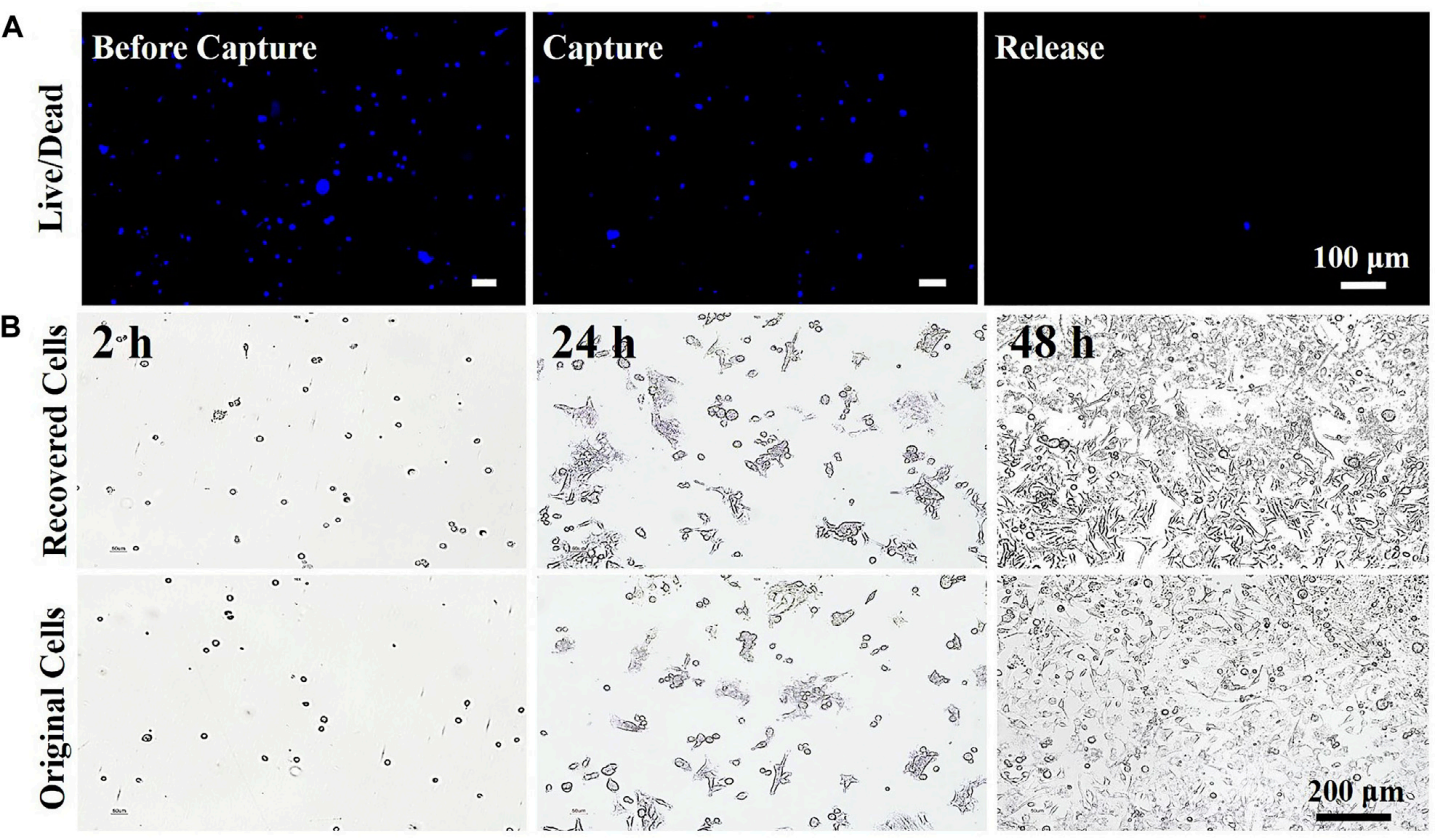
Microscope image of 4T1 cells after three cycles of capture and release. **(A)** Live/dead cell-staining images and **(B)** cell adhesive status and proliferation profiles of the original and recovered 4T1 cells over 3 days.

The viability of 4T1 cells during the capture/release cycle was studied using Hoechst and PI staining assays. From the fluorescence microscope image in Figure 8A, the cell release rate was determined to be approximately 91.9%, and the captured cells were completely released. We also found that there were no significant differences in cell viability during the capture/release cycle. The long-term cell culture and proliferation ability of the recovered 4T1 cells were also studied. The proliferation test results are shown in Figure 8B. The 4T1 cells started to proliferate after 24 h, and there was no evident difference between cell adhesion and growth behavior. Therefore, it could be inferred that the recovery of CTCs without loss of bioactivity could be achieved using this method. The prepared biosensor is suitable for the detection of specific target cells and can release the captured cells in a nondestructive manner for further analysis.

### Detection of CTCs in Simulated Samples With Leukocyte

To examine the applicability of our method in a simulated sample, leukocyte samples spiked with 4T1 cells at concentrations of 1 ×10^2^, 1 ×10^3^, 1 ×10^4^, and 1 ×10^5^ cells/mL were tested using the proposed cytosensor, and the corresponding results are shown in Supplementary Table S3. The recovery results were consistent with the corresponding spiked amounts of 4T1 cells. These results reveal that the fabricated biosensor could resist leukocyte interference for highly sensitive and specific CTCs detection.

## Conclusion

In conclusion, the timely discovery and detection of CTCs is important for the early diagnosis and treatment of cancer. In the present study, the promising application prospects of AuNPs in the construction of biosensors for sensitive detection and noninvasive release of CTCs were demonstrated. To realize sensitive, specific detection of CTCs, an electrochemical cell sensor based on AuNPs was developed. The AuNPs were modified onto the surface of the electrode and then covalently bonded by anti-EpCAM for specific recognition of EpCAM-positive cells. The captured cells on the electrode influenced the hot electron transport efficiency, leading to increased R_ct_ values. The changes in R_ct_ values or current values varied with the logarithm of CTCs concentration, and a sensitive, specific electrochemical strategy for CTCs detection was then reperted. Furthermore, the size effect of the AuNPs was studied. As the size of the AuNPs increased, the specific surface area of the cytosensors decreased, which led to fewer cells being captured by the sensors per unit area. The cell sensor constructed based on 17 nm AuNPs had a wider linear detection range from 8.0 × 10 to 1.0 × 10^7^ cells/mL and a lower LOD of 50 cells/mL for the 4T1 cells. The constructed cytosensor released the captured cells in a nondestructive manner when the Gly-HCl eluent was introduced. The recovered cells could be of great significance for early screening, postoperative monitoring, and selection of personalized treatment schemes for cancer patients.

## Data Availability

The original contributions presented in the study are included in the article/Supplementary Material, further inquiries can be directed to the corresponding authors.
