# Supplementary material for: Multifunctional Gold Nano-Cytosensor With Quick Capture, Electrochemical Detection, and Non-Invasive Release of Circulating Tumor Cells for Early Cancer Treatment
Source: Front Bioeng Biotechnol. 2021 Nov 11;9:783661. doi: 10.3389/fbioe.2021.783661 (PMC8632441; doi:10.3389/fbioe.2021.783661)
Supplement: Supplementary file 1 [file DataSheet1.docx]

Supplementary Material

**Multifunctional Gold Nano-Cytosensor with Quick Capture，Electrochemical Detection，and Non-invasive Release of Circulating Tumor Cells for early cancer treatment**

**Rui Zhang ^1,2^, Qiannan You ^1,2^,** **Mingming Cheng ^1,2^, Mingfeng Ge ^2^, Qian Mei ^2^, Li Yang ^3,4*^, Wen-Fei Dong^2^ and Zhimin Chang ^2,5*^**

1 School of Biomedical Engineering (Suzhou), Division of Life Sciences and Medicine, University of Science and Technology of China, Hefei, China

2 CAS Key Laboratory of Biomedical Diagnostics, Suzhou Institute of Biomedical Engineering and Technology, Chinese Academy of Science (CAS), Suzhou, China

3 College of Life Science&Biotechinology, Mianyang Teachers’ College, Mianyang, China

4 Chongqing Institute of Green and Intelligent Technology, Chinese Academy of Sciences, Chongqing, China

5 Jinan Guokeyigong Science and Technology Development Co., Ltd, Jinan, China

Corresponding Author

*** Correspondence:**Corresponding Author
changzm@sibet.ac.cn; ylyhp@126.com

# Materials

Trisodium citrate, Roswell Park Memorial Institute-1640 (RPMI-1640) culture medium, penicillin-streptomycin solution, and fetal bovine serum albumin (FBS) were purchased from Adamas-beta. HAuCl_4_, Bovine Serum Albumin (BSA), Gly-HCl, mercaptoethylamine and Nafion perfluorinated resin solutions were purchased from Sigma-Aldrich. KCl, sodium citrate, NaOH, K_2_CO_3_, HCl, K_4_[Fe(CN)_6_] and K_3_[Fe(CN)_6_] were purchased from Sinopharm Chemical Reagent Co., Ltd. Mouse monoclonal anti-EpCAM antibodies were purchased from Hushi Medicine Technology Co., Ltd. (Shanghai, China). 4T1 cells (mouse breast cancer cells), J774A.1 cells (mouse macrophages), HeLa cells, Hoechst, propidium iodide (PI), and DAPI staining solution for live or dead cells were obtained from Beyotime Biotechnology Co., Ltd. Phosphate buffered saline (PBS, pH 7.4) was used as the rinsing solution. The working solution contained 5 mM [Fe(CN)_6_] ^3-/4-^ and 0.1 M KCl. Deionize water (18.2 MΩ) was used in all experiments. All chemicals were of reagent grade and used as received.

# Instrumentation

The electrochemical experiments were conducted using a chi660e electrochemical workstation. The traditional three-electrode system was used in all experiments, with a saturated AgCl electrode as the reference electrode, and a Pt electrode as the counter electrode. To analyze AuNPs and the modified electrodes, transmission electron microscopy (TEM) and scanning electron microscopy (SEM) were employed. X-ray photoelectron spectroscopy (XPS) was performed using an ESCALAB 250 (Thermo Electron Company). Fluorescent images of the cells were obtained from a Nikon inverted microscope (ECLIPSE TE-2000U) equipped with a video camera (DS-U1).

# Supplementary Figure Caption


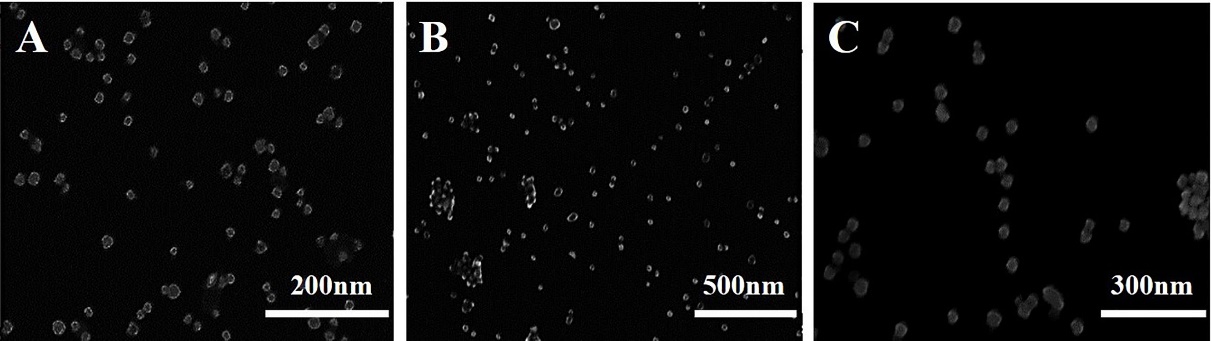


**Supplementary Figure 1.** The SEM images of the AuNPs/ GCE slide fabricated AuNPs by of different sizes. **(A)** G0/GCE，**(B)** G1/GCE，**(C)** G2/GCE.


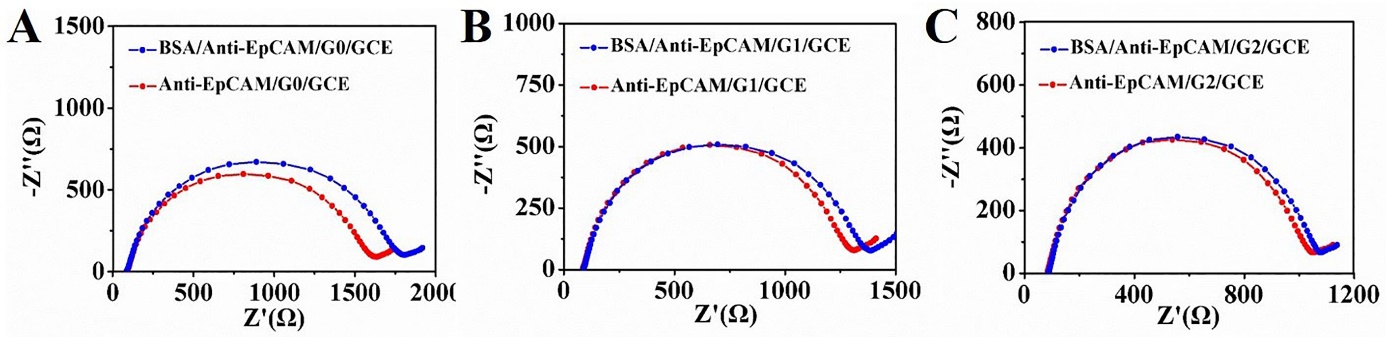


**Supplementary Figure 2.** The EIS curves of the cytosensors fabricated by AuNPs of different sizes. **(A)** The G0 cytosensor, **(B)** G1 cytosensor, **(C)** G2 cytosensor.


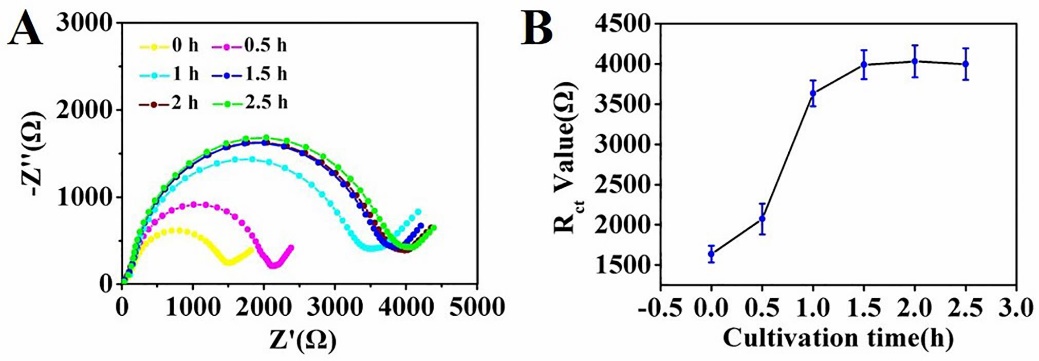


**Supplementary Figure 3.** The EIS curves **(A)** and R_ct_ value **(B)** of the cytosensors incubated with 4T1 cells (1.0 x 10^4^ cells/mL) for 0.5 h, 1 h, 1.5 h, 2 h and 2.5 h at 37 °C.


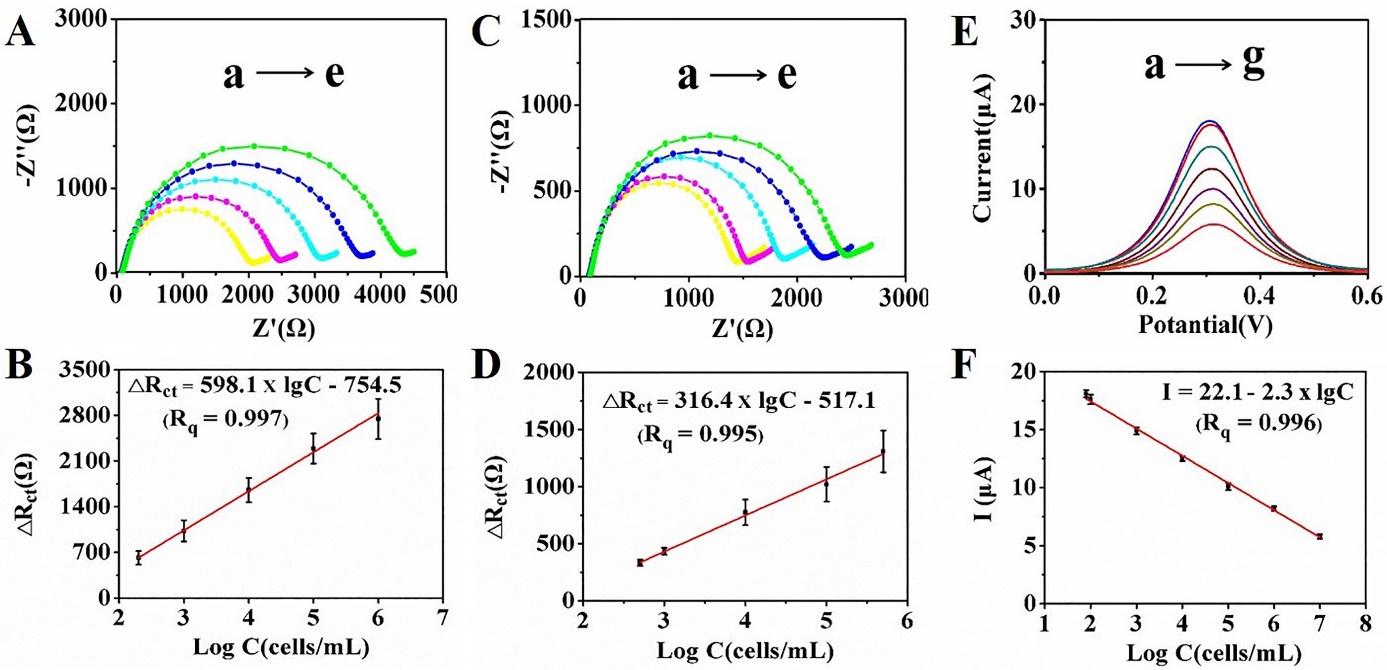


**Supplementary Figure 4.** The EIS or DPV curves of the cell sensors after capturing different concentrations of 4T1 cells. **(A)** The EIS curves of G1 cytosensor. **(B)** The linear relationship between impedance value of G1 cytosensor and the logarithm of cells concentration, (a) 2.0 × 10^2^, (b) 1.0 × 10^3^, (c) 1.0 × 10^4^, (d) 1.0 × 10^5^, (e) 1.0 × 10^6^ cells/ mL. **(C)** The EIS curves of G2 cytosensor. **(D)** The linear relationship between impedance value of G2 cytosensor and the logarithm of cells concentration, (a) 5.0 × 10^2^, (b) 1.0 × 10^3^, (c) 1.0 × 10^4^, (d) 1.0 × 10^5^, (e) 5.0 × 10^5^ cells/mL. **(E)** The DPV curves of G0 cytosensor. **(F)** The relationship between peak current value of G0 cytosensor and the logarithm of cells concentration, (a) 8.0 × 10, (b) 1.0 × 10^2^, (c) 1.0 × 10^3^, (d) 1.0 × 10^4^, (e) 1.0 × 10^5^, (f) 1.0 × 10^6^, (g) 1.0 × 10^7^ cells/mL.


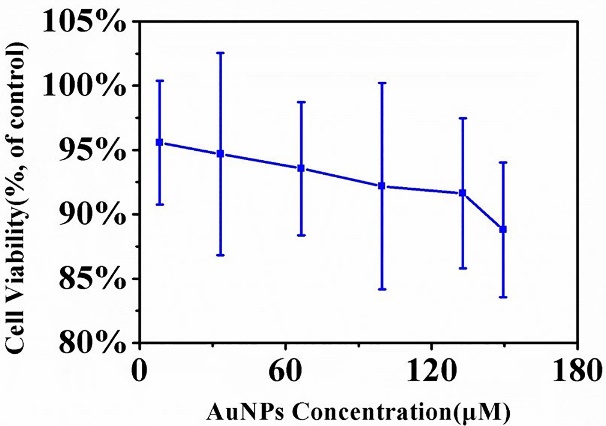


**Supplementary Figure 5.** Effect of AuNPs on the viability of 4T1 cells. Cell viability was determined by proposed WST-1 method. Error bars were the standard deviation of six replicate determinations.

**Supplementary Table 1:** Values of R_ct_ for the Stepwise Construction of the Cytosensor.

| **Assembly process** | **R_ct_(Ω)** | | | |
| --- | --- | --- | --- | --- |
|  |  | **G0** | **G1** | **G2** |
| Bare GCE | 456.7 |  |  |  |
| AuNPs |  | 25.8 | 122.4 | 319.9 |
| Anti-EpCAM |  | 1437.9 | 1149.7 | 909.9 |
| BSA |  | 1605.7 | 1216.1 | 940.4 |

**Supplementary Table 2.** Comparison of different cytosensors for determination of CTCs.

| **Cytosensor** | **Analyte** | **Detection**  **method** | **Linear range C/(cells/mL)** | **Detection Limit C/(cells/mL)** | **Reference** |
| --- | --- | --- | --- | --- | --- |
| PDA NPs/PGE | A-549 cells | EIS | 1.0 × 10^2^-1.0 × 10^5^ | 25 | Zhang et al., 2010; |
| Microfluidic chip | CCRF-CEM | Fluorescence | 4 × 10^2^ -5 × 10^6^ | 400 | Zhu et al., 2012; |
| Nanochannel-ionchannel  hybrid | CCRF-CEM | SWV | 1.0 × 10^2^-1.0 × 10^6^ | 100 | Cao et al., 2017; |
| PDCNx/TH^+^/AuNPs/GCE | Hela | EIS | 8.0 × 10^2^-2.0 × 10^7^ | 500 | Bolat et al., 2021 |

Notes: PDA NPs: Poly-dopamine nanoparticles. PGE: Pencil graphite electrode. PDCN_x_: Functionalized carbon nanotubes. TH^+^: Thionine chloride. GCE: Glassy carbon electrode.

**Supplementary Table 3:** Recoveries and reproducibility (indicated by relative standard deviation, EIS) of the spiked 4T1 cells detection by the proposed cytosensor in the simulated samples (n=5).

| **Spiked cells cells/mL** | **Detected cells**  **Cells/mL** | **Recoveries**  **(%)** | **Spiked Cells**  **Cells/mL** | **Detected cells**  **Cells/mL** | **Recoveries**  **(%)** |
| --- | --- | --- | --- | --- | --- |
| 100  100 | 98.2  102.4 | 98.20  102.40 | 1000  1000 | 1005  979 | 100.5  97.9 |
| 100  100 | 103.7  97.5 | 103.70  97.50 | 1000  1000 | 1002  997 | 100.1  99.7 |
| 100 | 99.2  **RSD%** | 99.20  2.71 | 1000 | 1006  **RSD%** | 100.6  1.11 |
| **Spiked cells cells/mL** | **Detected cells**  **Cells/mL** | **Recoveries**  **(%)** | **Spiked Cells**  **Cells/mL** | **Detected cells**  **Cells/mL** | **Recoveries**  **(%)** |
| 10000  10000 | 10038  9875 | 100.38  98.75 | 100000  100000 | 97580  103910 | 97.58  103.91 |
| 10000  10000 | 10152  9757 | 101.52  97.57 | 100000  100000 | 97463  103532 | 97.46  103.53 |
| 10000 | 10280  **RSD%** | 102.80  2.09 | 100000 | 102655  **RSD%** | 102.65  3.15 |

# References

Bolat, G., Vural, O.A., Yaman, Y.T., and Abaci, S. (2021). Polydopamine nanoparticles-assisted impedimetric sensor towards label-free lung cancer cell detection. *Materials Science and Engineering: C* 119**,** 111549.

Cao, J., Zhao, X.-P., Younis, M.R., Li, Z.-Q., Xia, X.-H., and Wang, C. (2017). Ultrasensitive capture, detection, and release of circulating tumor cells using a nanochannel–ion channel hybrid coupled with electrochemical detection technique. *Analytical chemistry* 89**,** 10957-10964.

Zhang, J.-J., Cheng, F.-F., Zheng, T.-T., and Zhu, J.-J. (2010). Design and implementation of electrochemical cytosensor for evaluation of cell surface carbohydrate and glycoprotein. *Analytical chemistry* 82**,** 3547-3555.

Zhu, J., Nguyen, T., Pei, R., Stojanovic, M., and Lin, Q. (2012). Specific capture and temperature-mediated release of cells in an aptamer-based microfluidic device. *Lab on a Chip* 12**,** 3504-3513.
